# Factors associated with laryngeal injury after intubation in children: a systematic review

**DOI:** 10.1007/s00405-024-08458-7

**Published:** 2024-02-08

**Authors:** L. L. Veder, K. F. M. Joosten, M. K. Timmerman, B. Pullens

**Affiliations:** 1 Department of Otorhinolaryngology, Erasmus Medical Center, Sophia Children’s Hospital, Room SP 1421a, Dr Molewaterplein 60, 3015 GJ Rotterdam, The Netherlands; 2 Department of Pediatrics, Intensive Care Unit, Erasmus Medical Center, Sophia Children’s Hospital, Rotterdam, The Netherlands

**Keywords:** Intubation injury, Laryngeal injury, Risk factors, Pediatric airway

## Abstract

**Purpose:**

The purpose of this study is to evaluate all potential factors associated with laryngeal injury after endotracheal intubation in the pediatric population.

**Methods:**

A systematic literature search was conducted in Medline, Embase, Cochrane, web of science and Google scholar up to 20th of March 2023. We included all unique articles focusing on factors possibly associated with intubation-injury in pediatric patients. Two independent reviewers determined which articles were relevant by coming to a consensus, quality of evidence was rated using GRADE criteria. All articles were critically appraised according to the PRISMA guidelines. The articles were categorized in four outcome measures: post-extubation stridor, post-extubation upper airway obstruction (UAO) necessitating treatment, laryngeal injury found at laryngoscopy and a diagnosed laryngotracheal stenosis (LTS).

**Results:**

A total of 24 articles with a total of 15.520 patients were included. The incidence of post-extubation stridor varied between 1.0 and 30.3%, of post-extubation UAO necessitating treatment between 1.2 and 39.6%, of laryngeal injury found at laryngoscopy between 34.9 to 97.0% and of a diagnosed LTS between 0 and 11.1%. Although the literature is limited and quality of evidence very low, the level of sedation and gastro-esophageal reflux are the only confirmed associated factors with post-extubation laryngeal injury. The relation with age, weight, gender, duration of intubation, multiple intubations, traumatic intubation, tube size, absence of air leak and infection remain unresolved. The remaining factors are not associated with intubation injury.

**Conclusion:**

We clarify the role of the potential factors associated with laryngeal injury after endotracheal intubation in the pediatric population.

**Supplementary Information:**

The online version contains supplementary material available at 10.1007/s00405-024-08458-7.

## Introduction

Typically, prematurely born neonates, newborns with congenital anomalies, children requiring ventilation during and after surgery and children suffering from serious infections or trauma, are the patients needing intubation. Intubation potentially results in laryngeal injury either through the act of intubation itself or the pressure exerted by the endotracheal tube [1–3].

Although most injuries will heal spontaneously after extubation, some children may develop a serious laryngeal stenosis with typical signs of severe upper airway obstruction (UAO) [4]. Depending on the degree of the obstructive symptoms, treatment ranges from non-surgical therapies such as administration of steroids, nebulizing epinephrine and/or oxygen therapy (e.g., high nasal oxygen flow), endoscopic surgical treatment or in severe cases the need for a tracheostomy and/or reconstruction of a laryngotracheal stenosis (LTS) [5, 6].

Only a vast minority of intubated children develop a LTS. In these patients, the cause is thought to be multifactorial but which factors contribute to the development of post-extubation laryngeal injuries and stenosis is not clear. Multiple contributing factors have been mentioned in the literature, including age, duration of intubation, multiple intubations, traumatic intubation, absence of air leak, the use of a cuffed tube and infection, but study results have been inconsistent [7–12]. Knowing these factors is important to prevent laryngeal injury as much as possible since they might develop into chronic lesions, like a LTS. We performed a systematic review to identify and evaluate all factors possibly contributing to post-extubation injury after endotracheal intubation in the pediatric and neonatal patients.

## Methods

### Inclusion criteria

Phase one of our review focused on identifying possibly associated factors for evaluation. We included all unique studies that answered our clinical question for the varying possibly associated factors, “Is ‘the concerning factor’ associated with the development of post-extubation laryngeal injury in the pediatric population?” Patient related factors as well as intervention related factors were reviewed. In case of patient related associated factors, our final PICO characteristics were: (P) pediatric patients younger than 18 years of age with the concerning factor; (I) endotracheal intubation; (C): not applicable; (O) Post-extubation laryngeal injury. In case of associated factors related to the intervention, our final PICO characteristics were: (P) pediatric patients younger than 18 years of age; (I) endotracheal intubation with the concerning factor; (C): not applicable (/ or: an uncuffed tube); (O) Post-extubation laryngeal injury.

### Information sources and search strategy

The search was carried out by a Biomedical Information Specialist of the Medical Library of the Erasmus Medical Center in the databases Medline ALL via Ovid (1946 to Daily Update), Embase.com (1971-present), Web of Science Core Collection (Science Citation Index Expanded (1975-present); Social Sciences Citation Index (1975-present); Arts & Humanities Citation Index (1975-present); Conference Proceedings Citation Index- Science (1990-present); Conference Proceedings Citation Index- Social Science & Humanities (1990-present) and Emerging Sources Citation Index (2015-present)) and the Cochrane Central Register of Controlled Trials via Wiley (1992-present). Additionally a search was performed in Google Scholar from which the 200 most relevant references were downloaded using the software Publish or Perish [13]. The search was limited to English language. Case reports, animal studies, articles focusing on adults and/ or congenital stenosis and articles that did not concern post-extubation injury and associated factors were excluded. See supplement 1. The search was performed on 04 May 2018 and updated on 20 March 2023. The results of the search strategies were uploaded into an Endnote library (Clarivate Analytics, Version 20.3) and duplicates were eliminated. Two reviewers (L.V., B.P.) independently screened title and abstract. Articles that did not meet the inclusion criteria were excluded. Any disagreements in this phase were resolved by consensus. After initial screening, the same two reviewers independently assessed the full text of the remaining articles for compliance with eligibility criteria. Discrepancies were settled through discussions.

### Data collection and management

The following data were extracted: (1) study design and setting, (2) number of patients, (3) description of characteristics of study groups, (4) factors associated with post-intubation laryngeal injury, 5) outcome definition, (6) duration of follow-up, (7) statistics.

We classified the associated factors into four categories: (a) confirmed factors were factors with a positive correlation, confirmed in all included studies, (b) unresolved factors were factors with inconsistent correlation in the included studies, (c) factors with no studies confirming any relation to post-extubation injury were classified as unrelated to intubation injury; (d) factors of which no literature is available were termed ‘unknown’.

### Certainty of evidence

The reporting of this systematic review was guided by the standards of the Preferred Reporting Items for Systematic Review and Meta-Analysis (PRISMA) Statement [14], see supplement 2a + 2b. Assessment of evidence quality and assessment risk of bias using was done by two independent reviewers (L.V., B.P.) using GRADE’s approach [15] and using the RoB 2 tool for randomized controlled trials (RCT) [16], the ROBINS-I tool for non-randomised studies of interventions [17] and the ROBINS-E tool for observational epidemiological studies [18]. A protocol was not prepared and the review was not registered in a database.

## Results

See Tables 1, 2, 3, 4 and 5, 6, 7, 8.

**Table 1:** Studies with outcome ‘post-extubation stridor’

Author	Study group	N	Stridor (%)	Treatment of UAO	Laryngoscopic findings	Incidence LTS	Study design	Follow-up	Statistics
Stridor neonates		227							
Da Silva et al. [21]	Neonates; Weight ≤ 1.5 kg	227	4.8	4.8%	n.a	0.4%	Prospective	Until discharge from the unit	Univariate analysis
Stridor children		2683							
De Jong et al. [22]	Children with down syndrome undergoing cardiac surgery	99	30.3	Not described	n.a	5.1% acquired 1.0% congenital	Retrospective	1 month-5 years	Multivariate analysis
Deakers et al. [24]	All children	188	14.9	Not described	n.a	0%	Prospective	18 months	Multivariate analysis
Veder et al. [12]	All Children ETT > 24 h	150	18.7	15.3%	n.a	0.7%	Prospective	Not described	Multivariate analysis
Weiss et al. [35]	Children Age < 5 yrs ETT: during surgery	2246	4.5	Not described	n.a	not described	RCT	1 h	Univariate analysis

*UAO* upper airway obstruction; *LTS* laryngotracheal stenosis; *ETT* endotracheal tube; *n.a.* not applicable; *RCT* randomized controlled trial

**Table 2:** Studies with outcome ‘treatment for post-extubation UAO’

Author	Study group	N	Stridor	Treatment of UAO	Laryngoscopic findings	Incidence LTS	Study design	Follow-up	Statistics
Treatment neonates		1710							
DeMichele et al. [25]	Neonates Weight < 5 kg Cardiac surgery	196	20.9%	20.5%	n.a	0%	Retrospective	not described	Multivariate analysis
Nicklaus et al. [10]	Neonates Weight < 1.5 kg	289	3.5%	19.4%	2.4% LTS	2.4%	Prospective	Mean 18 months (Range 6–36 months)	Univariate analysis
Suzumura et al. [11]	Neonates Age < 3 days ETT > 14 days	63	Not described	11.1%	11.1% LTS	11.1%	Not clear	Not described	Multivariate analysis
Williams et al. [36]	Neonates Weight < 5 kg After surgery	1162	Not described	not described	n.a	Not described	Retrospective	48 h	Univariate analysis
Treatment children		8313							
De Wit et al. [23]	Children Age < 7 years ETT: during surgery	6796	1.0%	not described	n.a	n.a	Retrospective	Not described	Multivariate analysis
Jorgensen et al. [8]	All children with bronchiolitis	144	Not described	39.6%	n.a	4.2%	Retrospective	Mean 9.3 months (Range 1–54 months)	Univariate analysis
Kemper et al. [9]	Children Age < 15 years ETT > 12 h Trauma/ burns	25	Not described	37.0%	n.a	Not described	Prospective	24–48 h after extubation	Multivariate analysis
Khine et al. [28]	Children Age < 8 years ETT: during surgery	488	2.7%	1.2%	n.a	0%	Prospective	Not described	Univariate analysis
Newth et al. [32]	All children	860	5.8%	5.8%	n.a	Not described	Prospective	During admission ICU	Univariate analysis

*UAO* upper airway obstruction; *LTS* laryngotracheal stenosis; *ETT* endotracheal tube; *n.a.* not applicable

**Table 3:** Studies with outcome ‘endoscopic confirmed post-extubation injury’

Author	Study group	N	Stridor	Treatment of UAO	Laryngoscopic findings	Incidence LTS	Study design	Follow-up	Statistics
Endoscopy neonates		227							
Albert et al. [19]	Neonates ETT > 24 h	30	30.0%	Not described	66.7% laryngeal abnormalities Rigid laryngoscopy at extubation	Not described	Prospective	Not described	Multivariate analysis
Fan et al. [26]	All neonates	95	Not described	Not described	43.2% moderate to major laryngeal injury Flexible laryngoscopy < 48 h at extubation and after ≥ 7 days	6.3%	Prospective	1–6 months	Multivariate analysis
Sherman et al. [33]	Neonates ETT > 7 days	102	n.a	n.a	9.8% moderate to severe lesions Flexible laryngoscopy 2–3 weeks after extubation	9.8%	Prospective	2–3 weeks	Multivariate analysis
Endoscopy children		480							
Bharti et al. [7]	Children 1–15 years ETT > 48 h	34	8.8%	5.9%	97.0% acute laryngeal injury (88.0% significant) Flexible laryngoscopy at extubation and after 3 – 4 weeks	5.9%	Prospective	3–4 weeks	Univariate analysis
Gomes Cordeiro et al. [27]	All children Weight ≥ 1250 gr	215	not described	Not described	34.9% moderate to severe lesions Rigid or flexible laryngoscopy at extubation	2.8%	Prospective	not described	Multivariate analysis
Manica et al. [30]	Children 28 days – 5 yrs ETT > 24 h	231	not described	Not described	44.2% moderate to severe lesions Flexible laryngoscopy within 8 h after extubation	Not described	Prospective	7–10 days	Multivariate analysis

*UAO* upper airway obstruction; *LTS* laryngotracheal stenosis; *ETT* endotracheal tube; *n.a.* not applicable

**Table 4:** Studies with outcome ‘confirmed laryngotracheal stenosis’

Author	Study group	*N*	Stridor	Treatment of UAO	Laryngoscopic findings	Incidence LTS	Study design	Follow-up	Statistics
LTS Neonates		196							
Lowery et al. [29]	Neonates with VAP Age < 2 yrs	120	n.a	n.a	n.a	7.5% total 8.3% VAP 6.7% non VAP	Retrospective	Not described	Univariate analysis
Thomas et al. [34]	Neonates Weight ≥ 3 kg Age < 3 months	76	13.7%	not described	n.a	0%	RCT	24 months	Univariate analysis
LTS children		1684							
Cakir et al. [20]	All children	112	n.a	n.a	n.a	n.a	Retrospective	Not described	Univariate analysis
Mossad et al. [31]	Children undergoing cardiac surgery	1572	n.a	n.a	n.a	1.1% (whole group) 2.3% (< 1 yrs) 2.1% (< 2 yrs)	Retrospective	Not described	Univariate analysis

*UAO* upper airway obstruction; *LTS* laryngotracheal stenosis; *ETT* endotracheal tube; *n.a.* not applicable; *VAP* ventilator associated pneumonia; *RCT* randomized controlled trial

**Table 5:** Factors associated with ‘post-extubation stridor’

Author	Sedation/ activity	Reflux	Age	Weight	Gender	Duration of intubation	Multiple intubations	Traumatic intubation	Tube size	Air leak	Infection	Gest. age	Skill level	Cuffed tube	Steroids	Syndrome/ comorbidity
Stridor neonates																
Da Silva et al. [21]				–			+	+	–	–	+	–			+	
Stridor children																
De Jong et al. [22]			+ (‘young age’)		–	–	+		–							
Deakers et al. [24]														–		
Veder et al. [12]			+ (‘young age’)			–		+	–		–		–	+ ^a^	–	–
Weiss et al. [35]														-		

^a^When using a cuffed tube in children aged between 0 and 1 year old

**Table 6:** Factors associated with ‘treatment for post-extubation upper airway obstruction’

Author	Sedation/ activity	Reflux	Age	Weight	Gender	Duration of intubation	Multiple intubations	Traumatic intubation	Tube size	Air leak	Infection	Gest. age	Skill level	Cuffed tube	Steroids	Syndrome/ comorbidity
Treatment neonates																
DeMichele et al. [25]			–	–		–			–			–			–	–^a^
Nicklaus et al. [10]		+		+	+ (girls)	+	+	+	–						+	
Suzumura et al. [11]				–		–	–		–		+ (tube < 14 days)	–				
Williams et al. [36]														–		
Treatment children																
De Wit et al. [23]														-		
Jorgensen et al. [8]			+ (> 12 months)		–	+ (< 3 days)				–		–			–	–
Kemper et al. [9]			–^a^			–		–	–	+						
Khine et al. [28]														–		
Newth et al. [32]														–		

^a^Significant in univariate analysis, not in multivariate analysis

**Table 7:** Factors associated with ‘endoscopic confirmed post-extubation injury’

Author	Sedation/ activity	Reflux	Age	Weight	Gender	Duration of intubation	Multiple intubations	Traumatic intubation	Tube size	Air leak	Infection	Gest. age	Skill level	Cuffed tube	Steroids	Syndrome/comorbidity
Endoscopy neonates																
Albert et al. [19]	+		+	-		^−^	-					-				
Fan et al. [26]						+ (≥ 7 days)	–^a^ (≥ 3)	–		–		–				
Sherman et al. [33]				-		+ (> 25 days)	+	-	+ ^b^			–				
Endoscopy children																
Bharti et al. [7]			–			–			–				–	–		
Gomes Cordeiro et al. [27]			- ^*a*^		–^a^ (boys)	–^a^	+	–	–							
Manica et al. [30]	+		-		-	-	+					–		–		

^a^Significant in univariate analysis, not in multivariate analysis

^b^When standardized to gestational age

**Table 8:** Factors associated with ‘a confirmed laryngotracheal stenosis’

Author	Sedation/ activity	Reflux	Age	Weight	Gender	Duration of intubation	Multiple intubations	Traumatic intubation	Tube size	Air leak	Infection	Gest. age	Skill level	Cuffed tube	Steroids	Syndrome/comorbidity
LTS neonates																
Lowery et al. [29]				–	–	–	–				-	–				-
Thomas et al. [34]														–		
LTS children																
Cakir et al. [20]			–	–	–	+	-					–				
Mossad et al. [31]			+ (< 2 yrs)			+ (> 48 h)								–		

**Fig. 1:**
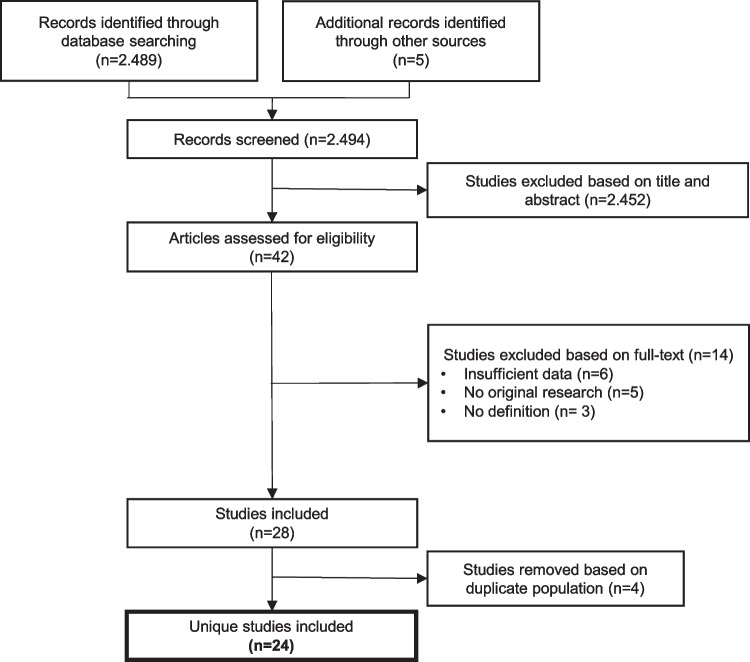
Included studies

A total of 2660 unique studies were identified. Another five relevant reports were found after checking reference lists. After reviewing title and abstract 42 potentially relevant studies remained. After full text screening 14 articles were excluded because of insufficient data, the study concerned no original research, or there was no clear outcome definition. Also, four articles were excluded with possible overlapping participants. Therefore, 24 unique studies [7–12, 19–36] with a total of 15,520 patients met the full eligibility criteria and were included in this review. See Fig. 1. In these studies we found 16 concerning factors to be evaluated. Eight patient related factors, namely under-sedation, presence of gastro-esophageal reflux, younger age, lower weight, gender, presence of infection, underlying comorbidity and shock. And eight intervention related factors, namely prolonged intubation, multiple intubations, traumatic intubations, a larger tube size than corrected for age, absence of air leak, a less skilled intubator, the use of a cuffed tube and use of steroids. For one factor (cuffed/non-cuffed tubes), a direct comparison could be made. All other factors were evaluated for their possible association with post intubation injury. Of these studies, two studies were RCT’s [34, 35], one non-randomized interventional trial [28], 12 prospective observational studies [7, 9, 10, 12, 19, 21, 24, 26, 27, 30, 32, 33] and 8 retrospective observational studies [8, 20, 22, 23, 25, 29, 31, 36]. For one study, the design was not clear [11].

The included articles were subsequently categorized in one of four predefined outcome measures: a) post-extubation stridor, b) direct treatment for all post-extubation UAO, c) endoscopic confirmed lesions or d) a diagnosed LTS.

### Outcome measures

#### Post-extubation stridor

Table 1 describes the study characteristics for the included studies who reported on post-extubation stridor as an outcome measure for laryngeal injury. The reported incidence of post-extubation stridor in the studies that used stridor as an outcome measure varied broadly from 4.5 to 30.3%. There was a lower incidence of post-extubation stridor after short duration of intubation (1.0% and 4.5%) [23, 35], a higher incidence of post-extubation stridor was found in children with trisomy 21 undergoing cardiovascular surgery (30.3%) [22] and in infants weighing less than 5 kg operated for congenital heart defects (20.9%) [25]. In neonates intubated for more than 24 h with endotracheal Coles tubes an incidence of stridor of 30.0% was reported [19]. The remaining studies reported an incidence varying between 1.2% and 18.7%.

#### Treatment for post-extubation UAO

Table 2 describes the study characteristics for the included studies who reported on treatment for post-extubation UAO as an outcome measure for laryngeal injury. Treatment of post-extubation UAO included treatment of all respiratory complications like stridor, retractions, respiratory distress, dyspnea, wheezing, with the use of corticosteroids, racemic epinephrine, respiratory support with high flow oxygen (Optiflow ®), reintubation, or the need for microscopic laryngeal surgery. The incidence of post-extubation UAO necessitating treatment varied widely between 4.8% and 39.6%.

#### Endoscopic confirmed lesions

Table 3 describes the study characteristics for the included studies who reported on endoscopic confirmed lesions as an outcome measure for laryngeal injury. Endoscopy was done in all children within 2 days after extubation with either flexible or rigid endoscopes and revealed abnormalities of the larynx in 34.9% to 97.0% of all patients, mostly edema and erythema. Significant lesions were reported in up to 88.0% of patients. Three studies repeated endoscopy two to three weeks later and showed persisting moderate to severe obstruction in 9.8–11.8% of patients [7, 26, 33].

#### Confirmed LTS

Table 4 describes the study characteristics for the included studies who reported on endoscopic confirmed LTS as an outcome measure for laryngeal injury. Overall, the incidence of a LTS varied between 0% and 11.1%.

### Factors and their relation with intubation injury.

#### Confirmed factors

#### Sedation

Two studies [19, 30] (261 patients) looked at the level of sedation, observed by activity scores recorded by the nurse [19], or documented by the need for extra doses of sedation [30]. Both studies showed that under-sedation was found to have a significant relationship with endoscopic confirmed moderate to severe laryngeal injury (seen in 44.2–66.7% of the patients) in multivariate analysis. The level of evidence of factor ‘sedation’ comes from two prospective observational studies. Due to a critical risk of bias, mainly by the risk of selection bias and of bias of misclassification of exposure, this was modified downward to a very low level of evidence.

#### Gastro-esophageal reflux

Only one prospective observational study of Nicklaus et al. [10] (289 patients) looked at reflux as an associated factor of post-extubation injury. They found a significant relationship between reflux and neonates that needed treatment for post-extubation UAO and eventually developing a LTS in univariate analysis. Due to a critical risk of bias, mainly by confounding, selection bias and bias of misclassification of exposure this was modified downward to a very low level of evidence.

### Unresolved factors

#### Age

The factor ‘age’ was reviewed in 11 studies [7–9, 12, 19, 20, 22, 25, 27, 30, 31] (2808 patients), using all four outcome measures. Of these, 7 studies found a significant correlation of age and the presence of post-intubation laryngeal injury, while 4 studies found no significant relationship. The level of evidence of factor ‘age’ comes from prospective and retrospective observational studies. Due to conflicting results (inconsistency) and a critical risk of bias, mainly by confounding and selection bias this was modified downward resulting in a very low level of evidence.

#### Weight

Eight studies [10, 11, 19–21, 25, 29, 33] (1139 patients) looked at weight at intubation as a contributing factor for laryngeal injury. Only one study of Nicklaus et al. [10] found a significant association with the development of a LTS in neonates with a very low birthweight. None of the remaining studies showed a significant association with weight at intubation in one of the outcome measures. The level of evidence of factor ‘weight’ comes from prospective and retrospective observational studies. The level of evidence was downgraded due to a critical risk of bias, mainly by confounding and selection bias and resulted in a level of evidence of very low.

#### Gender

Seven studies [8, 10, 20, 22, 27, 29, 30] (1210 patients) looked at the relationship between gender and post-extubation laryngeal injury. Nicklaus et al. [10] showed significant more girls that had to be treated for post-extubation UAO and Gomes Cordeiro et al. [27] found a significant relationship between boys and laryngeal injury seen at endoscopy in univariate analysis, but not in multivariate analysis. The remaining five studies found no association between gender and post-extubation injury. The level of evidence of factor ‘gender’ comes from seven prospective and retrospective observational studies. Due to conflicting results (inconsistency) and a critical risk of bias, mainly by confounding and selection bias, the level of evidence was downgraded to a very low level of evidence.

#### Duration of intubation

Sixteen studies [7–12, 19, 20, 22, 25–27, 29–31, 33] (3477 patients) assessed the duration of intubation as factor associated with laryngeal injury. Five studies [10, 20, 26, 31, 33] (2170 patients) found a longer duration of intubation to be a significant related factor. One study of Jorgenson et al. [8] showed discrepant results, with an increased risk for treatment of UAO when intubated less than three days in children intubated for bronchiolitis. The remaining nine studies [7, 9, 11, 12, 19, 22, 25, 29, 30] did not find any significant relationship for duration of intubation and one of the outcome measures, although Suzumura et al. [11] did find a clear difference in the incidence of LTS in neonates intubated less than 14 days (0%) versus intubated 14 days or more (11.2%). The level of evidence of factor ‘duration of intubation’ comes from prospective and retrospective observational studies. Due to conflicting results (inconsistency) and a critical risk of bias, mainly by confounding and selection bias, the level of evidence was downgraded resulting in a level of evidence of very low.

#### Multiple intubations

Eleven studies [10, 11, 19–22, 26, 27, 29, 30, 33] (1583 patients) looked at ‘multiple intubations’, ‘tube repositioning’, ‘reintubation’ or ‘tube exchange’ and its’ relation with post-extubation laryngeal injury. Most articles lack a clear definition, the number of intubations and/ or the range of intubations. Six studies [10, 21, 22, 27, 30, 33] showed a significant positive association. The remaining four studies [11, 19, 20, 29] showed no relation with multiple intubations and post-extubation laryngeal injury. The level of evidence of factor ‘multiple intubations’ comes from prospective and retrospective observational studies. Due to conflicting results (inconsistency) and a critical risk of bias, mainly by confounding, selection bias and bias of misclassification of exposure, the level of evidence was downgraded resulting in a level of evidence of very low.

#### Traumatic intubation

Seven studies [9, 10, 12, 21, 26, 27, 33] (1103 patients) investigated traumatic intubation as a factor related to post-extubation injury. The procedure was noted as traumatic if the procedure was described as traumatic by the physician who performed the intubation [10, 24], if a tube larger than corrected for age was used [24], if the intubation caused bleeding [10, 21], if several attempts were made, the tube passed through the larynx with difficulty [26], when a smaller tube was required because a larger tube would not pass [26] or if an emergency intubation took place in the field [9, 12]. Three studies [10, 12, 21] (666 patients) found a significant association with traumatic intubation. The remaining four studies did not find any relation. The level of evidence of factor ‘traumatic intubation’ comes from prospective observational studies. Due to conflicting results (inconsistency) and a critical risk of bias, mainly by confounding and selection bias, the level of evidence was downgraded resulting in a level of evidence of very low.

#### Tube size

Ten studies [7, 9–12, 21, 22, 25, 27, 33] (1364 patients) looked at tube size as a factor associated with post-extubation injury. Only Sherman et al. [33] found a significant association between tubes that are too large, standardized to gestational age, and endoscopic confirmed laryngeal injury. The level of evidence of factor ‘tube size’ comes from prospective and retrospective observational studies. Due to conflicting results (inconsistency) and a critical risk of bias, mainly by confounding and selection bias, the level of evidence was downgraded resulting in a level of evidence of very low.

#### Absence of air leak

Four studies [8, 9, 21, 26] (491 patients) investigated post-extubation injury and air leak. Kemper et al. [9] found a significant relation between the absence of air leak just before extubation and the need for treatment of post-extubation UAO in pediatric trauma patients. The other studies did not find an association between the absence of air leak and post-extubation laryngeal injury [8, 21, 26]. The level of evidence of factor ‘tube size’ comes from prospective and retrospective observational studies. Due to conflicting results (inconsistency), low number of included patients (imprecision) and a critical risk of bias, mainly by confounding and selection bias, the level of evidence was downgraded. This resulted in a level of evidence of very low.

#### Infection

Four studies [11, 12, 21, 29] (560 patients) examined the relation between post-extubation laryngeal injury and infection, which was stated as a (ventilator associated) pneumonia, bacterial tracheitis, bacterial colonization of the tube, respiratory infection, RSV status, sepsis, meningitis, or arthritis. Two studies showed a positive association between bacterial colonization of the tube and post-extubation stridor in neonates with a very low birthweight [21] and between an infection occurring within 14 days of intubation and a LTS in neonates [11]. The remaining studies did not find a significant association. The level of evidence of factor infection comes from prospective and retrospective observational studies. Due to conflicting results (inconsistency) and a critical risk of bias, mainly by confounding and selection bias, the level of evidence was downgraded resulting in a level of evidence of very low.

### Unrelated factors

For the factors ‘gestational age’, ‘skill level of intubator’, ‘cuffed tubes’, ‘steroids’ and ‘underlying comorbidity’ no significant factors were found in the various included studies. A more detailed description for these studies is given in the supplemental information.

### Unknown factors

No studies were found regarding post-extubation laryngeal injury and shock.

## Discussion

With this systematic review we aimed to give an overview of laryngeal injury after endotracheal intubation in children and to clarify the contribution of previous assumed associated factors. Obviously, all included studies are very heterogeneous. In an attempt to structure our findings, we divided the outcome measure in four categories (incidence): post-extubation stridor (1%–30.3%), the need for treatment of post-extubation UAO (5.4–39.6%), endoscopic confirmed laryngeal findings (34.9–97.0%) and a diagnosed LTS (0–11.1%).

As expected, the incidence of endoscopically confirmed laryngeal injury was highest. This confirms the general idea that nearly all intubations will cause some degree of laryngeal injury, but the clinical consequence of that damage varies widely between patients. This is an important finding when reviewing our results: factors contributing to early laryngeal injury are not necessarily important for the development of a life-threatening LTS. On the other hand, exclusion of a risk factor for early laryngeal injury may not necessarily prevent late laryngeal injury.

We found ‘the level of sedation’ and ‘gastro-esophageal reflux’ as the only confirmed associated factors with post-extubation laryngeal injury, although it must me noted that, concerning these factors, literature is limited and the quality of evidence is very low. Optimal analgesia/ sedation leads to a comfortable intubated patient, with no signs of distress, restlessness, agitation, or pain, but also no signs of excessive sedation. The degree of comfort can be determined by different scoring systems or by the need for additional sedation. Two studies showed a significant relationship with moderate to severe endoscopic confirmed lesions and under-sedation, observed by activity scores recorded by the nurse, or documented by the need for extra doses of sedation [19, 30].

Only one study looked at gastro-esophageal reflux [10]. In this study in univariate analysis a significant association between the presence of reflux and the need for treatment of post-extubation UAO was found. It was not specified how the diagnosis was established. Besides, due to the lack of supporting evidence, one should be cautious in drawing definite conclusions. If indeed presence of reflux is a factor for post-intubation laryngeal injury, both non-pharmacological as pharmacological treatment to prevent post-intubation sequelae should be considered.

Given the common agreement on the pathogenesis of intubation injury being exerted pressure of the tube on the airway structures, it is commonly accepted that factors like ‘duration of intubation’ and ‘the use of an oversized tube’ are associated with post-extubation laryngeal injury. It is conceivable that the mechanical pressure of the tube on mucosa, submucosa and deeper structures only is reversible if present for a limited time, like a few hours, but has an increased risk of chronic lesions and subsequent permanent scar tissue development after prolonged intubation. However, the factors ‘duration of intubation’, ‘multiple intubations’, ‘traumatic intubation’, ‘tube size’ and ‘absence of air leak’, but also the factors ‘age’, ‘weight’, ‘gender’, and ‘infection’ are all unresolved associated factors, with conflicting results and weak evidence in different patient groups and for different outcome measures. Therefore, no conclusion can be drawn for these factors. The relation with laryngeal injury is especially doubtful for the factors weight and tube size, since no studies except one showed any evidence for an association with post-extubation injury.

There was no evidence for an association between post-extubation laryngeal injury and the presumed factors gestational age, skill level of the physician who performed the intubation, the use of modern-day cuffed tubes (high-volume, low-pressure cuffed tubes), the use of steroids or underlying comorbidity. However, we do stress that this does not acquit the clinician from adhering to common best practices like selecting correct tube sizes, adhering to the correct cuff protocol and sufficient skill level of the intubator. The use of a cuffed tube has been a subject for debate for many years. With the development of the polyvinyl chloride high-volume, low-pressure cuffed endotracheal tubes and the introduction of the ultrathin polyurethane ‘Microcuff®’ pediatric endotracheal tubes, there has been an increase in the use of cuffed tubes in children from birth. The included studies, including neonates weighing less than three kilograms, did not show any relationship between the use of these tubes and post-extubation laryngeal injury, but these findings need to be confirmed in a large multicenter trial, with a long-term follow-up. The advantages of a cuffed tube, like the decrease in amount of tube changes and a better sealed airway [35], make the use of a cuffed tube favorable in certain circumstances.

The results of our review cannot resolve the debate on the use of steroids prior to extubation, unfortunately. While steroids are thought to protect against laryngeal injury, two studies showed an association between the higher use of steroids and an increase in post-extubation injury [10, 12]. This is interpreted as an inverse association, physicians use steroids in patients where difficulties at extubation are expected, not as a contributing factor to post-extubation injury.

Altogether, all studies which met our inclusion criteria have a very low quality of evidence and form an extremely heterogeneous group, varying considerably in several aspects, like the studied patient groups, outcome measures, study design, definitions of associated factors and follow-up period. The different outcome measures to detect post-extubation laryngeal injury have their own drawbacks; for instance, the outcome measure ‘awake flexible laryngoscopy’ can be used to identify glottic and possibly direct subglottic lesions, but it does not exclude all features of subglottic or tracheal damage. A similar point of discussion arises on the outcome measures: ‘post-extubation stridor’ and ‘treatment for UAO’, both of which are possibly not very specific for post-extubation laryngeal injury.

We excluded studies concerning congenital LTS but we did include one study [22] involving children with trisomy 21 in whom subglottic narrowing is a common clinical feature. These children possibly had a higher incidence of post-extubation stridor due to a congenitally narrower airway. A causal relationship between the higher incidence of post-extubation stridor and a congenital subglottic narrowing is possible but purely speculative in this group. One has to take into account that post-extubation stridor can also be caused by a vocal cord paralysis in these children after cardiac or thoracic surgery.

Over time, ongoing developments in the neonatal and pediatric care unit (e.g. high nasal oxygen flow) have led to fewer tracheal intubations and to the use of different endotracheal tubes (e.g. high-volume, low-pressure cuffed tubes). Besides, improvements in endoscopic surgical options for treating post-extubation laryngeal injury (e.g. balloon dilatation, cricoid split, intra-lesional steroids) have possibly led to a decrease in the development of LTS [6]. Since our included studies span almost 30 years, it is unclear what influence these developments have on our study results.

There are some important limitations in matching the included studies. The main limitation is the heterogeneity of the studies, which makes pooling the results in order to perform a meta-analysis not feasible and therefore we are unable to draw definitive conclusions for factors with conflicting results in the literature. Furthermore, ongoing developments in both the neonatal and pediatric intensive care unit and in improvements in endoscopic surgical options might have made older studies less relevant. To associate late laryngeal injury with risk factors of intubation, a follow-up period including repeated (flexible and rigid) endoscopy of at least one year, would be ideal. Also, with special attention for gastro-esophageal reflux and shock, generally considered to contribute to laryngeal injury.

In conclusion, despite the extensive literature search with a large number of patients included, there is still no convincing evidence regarding the relevance of different factors involved in the development of post-extubation laryngeal injury. With a very low quality of evidence, the level of sedation and gastro-esophageal reflux appears to be associated with laryngeal injury, making adequate levels of sedation extra relevant and treatment of anti-reflux justified. When attending good clinical practice, the factors gestational age, skill level of the intubator, the use of modern-day cuffed tubes and the use of steroids or underlying comorbidity do not have an association with post-extubation laryngeal injury.

### Supplementary Information

Below is the link to the electronic supplementary material.

Supplementary file1 (PDF 191 KB)

Supplementary file2 (PDF 341 KB)

Supplementary file3 (PDF 285 KB)

Supplementary file4 (DOCX 21 KB)

## Data Availability

Not applicable.
