# Supplementary material for: Factors associated with laryngeal injury after intubation in children: a systematic review
Source: Eur Arch Otorhinolaryngol. 2024 Feb 8;281(6):2833–47. doi: 10.1007/s00405-024-08458-7 (PMC11065910; doi:10.1007/s00405-024-08458-7)
Supplement: Supplementary file 1 — Supplementary file1 (PDF 191 KB) [file 405_2024_8458_MOESM1_ESM.pdf]

Supplement 1. Literature search.

embase.com

('endotracheal tube'/exp OR 'respiratory tract intubation'/exp OR 'laryngeal tube'/de OR 'artificial ventilation'/de OR 'extubation'/de OR 'intubation'/de OR (((endotrach* OR laryn* OR endobronch* OR nasotrach* OR trach* OR intratrach* OR stridor* OR subglott* OR glotti* OR vocal-cord*) NEAR/6 (tube OR tubes OR intubat* OR detubat* OR extubat* OR postintubat* OR postextubat* OR ventilat*)) OR (artificial* NEAR/3 (ventilat* OR respirat*))):ab,ti) AND ('larynx injury'/exp OR 'larynx stenosis'/de OR 'subglottic stenosis'/de OR 'stridor'/de OR 'larynx disorder'/de/dm_co OR 'larynx granuloma'/de OR (((laryn* OR subglott* OR glotti* OR vocal-cord*) NEAR/3 (injur* OR damage* OR trauma* OR stenos* OR sequel* OR adverse* OR lesion* OR tear* OR complicat* OR rupture* OR granulom*)) OR stridor* OR Laryngostenos*):ab,ti) AND (child/exp OR adolescent/exp OR adolescence/exp OR 'child behavior'/de OR 'child parent relation'/de OR pediatrics/exp OR childhood/exp OR 'child nutrition'/de OR 'infant nutrition'/exp OR 'child welfare'/de OR 'child abuse'/de OR 'child advocacy'/de OR 'child development'/de OR 'child growth'/de OR 'child health'/de OR 'child health care'/exp OR 'child care'/exp OR 'childhood disease'/exp OR 'child death'/de OR 'child psychiatry'/de OR 'child psychology'/de OR 'pediatric ward'/de OR 'pediatric hospital'/de OR 'pediatric anesthesia'/de OR 'pediatric intensive care unit'/de OR 'neonatal intensive care unit'/de OR (adolescen* OR preadolescen* OR infan* OR newborn* OR (new NEXT/1 born*) OR baby OR babies OR neonat* OR child* OR kid OR kids OR toddler* OR teen* OR boy* OR girl* OR minors OR underag* OR (under NEXT/1 (age* OR aging)) OR juvenil* OR youth* OR kindergar* OR puber* OR pubescen* OR prepubescen* OR prepubert* OR pediatric* OR paediatric* OR school* OR preschool* OR highschool* OR nicu OR picu):ab,ti) NOT (([Conference Abstract]/lim AND [1800-2016]/py) OR [Letter]/lim OR [Note]/lim OR [Editorial]/lim) AND [english]/lim NOT ([animals]/lim NOT [humans]/lim) NOT ('case report'/de OR ((case NEAR/3 report*)):ab,ti)

Medline Ovid

(Intubation, Intratracheal/ OR Airway Extubation/ OR laryngeal tube/ OR Respiration, Artificial/ OR (((endotrach* OR laryn* OR endobronch* OR nasotrach* OR trach* OR intratrach* OR stridor* OR subglott* OR glotti* OR vocal-cord*) ADJ6 (tube OR tubes OR intubat* OR detubat* OR extubat* OR postintubat* OR postextubat* OR ventilat*)) OR (artificial* ADJ3 (ventilat* OR respirat*))).ab,ti.) AND (larynx/in OR Laryngostenosis/ OR Laryngeal Diseases/co OR Granuloma, Laryngeal/ OR (((laryn* OR subglott* OR glotti* OR vocal-cord*) ADJ3 (injur* OR damage* OR trauma* OR stenos* OR sequel* OR adverse* OR lesion* OR tear* OR complicat* OR rupture* OR granulom*)) OR stridor* OR Laryngostenos*).ab,ti.) AND (exp Child/ OR exp Infant/ OR exp Adolescent/ OR exp "Child Behavior"/ OR exp "Parent Child Relations"/ OR exp "Pediatrics"/ OR "Child Nutrition Sciences"/ OR "Infant nutritional physiological phenomena"/ OR exp "Child Welfare"/ OR "Child Development"/ OR exp "Child Health Services"/ OR exp "Child Care"/ OR "Child Rearing"/ OR exp "Child development Disorders, Pervasive"/ OR "Child Psychiatry"/ OR "Child Psychology"/ OR "Hospitals, Pediatric"/ OR exp "Intensive Care Units, Pediatric"/ OR (adolescen* OR preadolescen* OR infan* OR newborn* OR (new ADJ born*) OR baby OR babies OR neonat* OR child* OR kid OR kids OR toddler* OR teen* OR boy* OR girl* OR minors OR underag* OR (under ADJ (age* OR aging)) OR juvenil* OR youth* OR kindergar* OR puber* OR pubescen* OR prepubescen* OR prepubert* OR pediatric* OR paediatric* OR school* OR preschool* OR highschool* OR nicu OR picu).ab,ti.) NOT (letter* OR news OR comment* OR editorial* OR congres* OR abstract* OR book* OR chapter* OR dissertation abstract*).pt. AND english.la. NOT (exp animals/ NOT humans/) NOT (case reports/ OR ((case ADJ3 report*)).ab,ti.)

Cochrane CENTRAL

((((endotrach* OR laryn* OR endobronch* OR nasotrach* OR trach* OR intratrach* OR stridor* OR subglott* OR glotti* OR vocal-cord*) NEAR/6 (tube OR tubes OR intubat* OR detubat* OR extubat* OR postintubat* OR postextubat* OR ventilat*)) OR (artificial* NEAR/3 (ventilat* OR respirat*))):ab,ti) AND ((((laryn* OR subglott* OR glotti* OR vocal-cord*) NEAR/3 (injur* OR damage* OR trauma* OR stenos* OR sequel* OR adverse* OR lesion* OR tear* OR complicat* OR rupture* OR granulom*)) OR stridor* OR Laryngostenos*):ab,ti) AND ((adolescen* OR preadolescen* OR infan* OR newborn* OR (new NEXT/1 born*) OR baby OR babies OR neonat* OR child* OR kid OR kids OR toddler* OR teen* OR boy* OR girl* OR minors OR underag* OR (under NEXT/1 (age* OR aging)) OR juvenil* OR youth* OR kindergar* OR puber* OR pubescen* OR prepubescen* OR prepubert* OR pediatric* OR paediatric* OR school* OR preschool* OR highschool* OR nicu OR picu):ab,ti)

Web of science

TS=(((((endotrach* OR laryn* OR endobronch* OR nasotrach* OR trach* OR intratrach* OR stridor* OR subglott* OR glotti* OR vocal-cord*) NEAR/5 (tube OR tubes OR intubat* OR detubat* OR extubat* OR postintubat* OR postextubat* OR ventilat*)) OR (artificial* NEAR/2 (ventilat* OR respirat*)))) AND ((((laryn* OR subglott* OR glotti* OR vocal-cord*) NEAR/2 (injur* OR damage* OR trauma* OR stenos* OR sequel* OR adverse* OR lesion* OR tear* OR complicat* OR rupture* OR granulom*)) OR stridor* OR Laryngostenos*)) AND ((adolescen* OR preadolescen* OR infan* OR newborn* OR (new NEAR/1 born*) OR baby OR babies OR neonat* OR child* OR kid OR kids OR toddler* OR teen* OR boy* OR girl* OR minors OR underag* OR (under NEAR/1 (age* OR aging)) OR juvenil* OR youth* OR kindergar* OR puber* OR pubescen* OR prepubescen* OR prepubert* OR pediatric* OR paediatric* OR school* OR preschool* OR highschool* OR nicu OR picu))) AND DT=(article) AND LA=(english)

Google scholar

"endotracheal|laryngeal|tracheal tube|tubes|intubation|extubation"|"artificial ventilat|respiration"
"laryngeal|subglottic|glottic injuries|injury|damage|stenosis"
adolescents|infants|newborns|newborn|neonate|neonatal|children|pediatric
