# Supplementary material for: Factors associated with laryngeal injury after intubation in children: a systematic review
Source: Eur Arch Otorhinolaryngol. 2024 Feb 8;281(6):2833–47. doi: 10.1007/s00405-024-08458-7 (PMC11065910; doi:10.1007/s00405-024-08458-7)
Supplement: Supplementary file 4 — Supplementary file4 (DOCX 21 KB) [file 405_2024_8458_MOESM4_ESM.docx]

**Supplemental information unrelated factors**

*Unrelated factors.*

*Gestational age*. Ten studies [8, 11, 19-21, 25, 26, 29, 30, 33] (917 patients), including seven studies in premature and mature neonates, looked at gestational age and their relation with post-extubation injury. None of these studies found an association with post-extubation injury. The level of evidence of factor ‘gestational age’ comes from prospective and retrospective observational studies. Due to a critical risk of bias, mainly by confounding and selection bias, the level of evidence was downgraded resulting in a level of evidence of very low.

*Skill level*. Two studies [7, 12] (184 patients) looked at the skill level of the intubator and post-extubation stridor and endoscopic confirmed lesions. Both did not find an association between the skill level of the intubator and post-extubation lesions. The level of evidence of factor ‘skill level’ comes from two prospective observational studies. Due to low number of included patients (imprecision) and a critical risk of bias, mainly by confounding and selection bias, the level of evidence was downgraded to a very low level of evidence.

*Cuff*. Eleven studies [7, 12, 23, 24, 28, 30-32, 34-36] (13.803 patients) looked at the relationship between the use of a cuffed tube and post-extubation laryngeal injury. The manufacturers of the tubes used in the studies differed and were not clearly mentioned in all studies. When mentioned, tubes used were Portex, Mallinckrodt, Microcuff, Rüsch, Sheridan and Shiley. Veder et al. [12] found in a prospective observational study a significant higher amount of post extubation stridor using a cuffed tube in children under the age of one year. They used the older, high-pressure, low-volume type cuffed tubes. All other studies, among them studies including the high-volume, low-pressure cuffed tubes and among them two studies included neonates [34, 36] did not find a significant relationship. Two RCT’s [34, 35] (2.322 patients), one non-randomized interventional trial (488 patients) [28], 5 prospective observational studies [7, 12, 24, 30, 32] (1.462 patients) and 3 retrospective observational studies [23, 31, 36] (9.530 patients) were included in this review. The level of evidence was decided based on the two RCT’s and the non-randomized trial. Due to inadequate allocation concealment, the level of evidence was downgraded to a moderate level of evidence. The other, lower quality studies on this subject support the conclusion.

*Steroids.* Five studies [8, 10, 12, 21, 25] (1.006 patients) looked at the association between the use of steroids and post-extubation injury. Two studies showed a significant relationship between the higher use of steroids and post-extubation stridor [12] and the need for post-extubation treatment in neonates with a very low birthweight [10]. The remaining three studies did not show any association. The level of evidence of factor ‘steroids’ comes from prospective and retrospective observational studies. Due to a critical risk of bias, mainly by confounding and selection bias, the level of evidence was downgraded resulting in a level of evidence of very low.

*Underlying comorbidity.* Four studies [8, 12, 25, 29] (610 patients) looked at the relationship between underlying comorbidity and post-extubation laryngeal injury. Underlying comorbidity was described as ‘syndromes’ or ‘significant medical history’. Only one study of DeMichele et al. [25] showed a positive association between significant comorbidity and the need for treatment of post-extubation UAO in neonates undergoing cardiac surgery in univariate analysis, but this did not remain after multivariate analysis. Also, the other studies showed no relation between underlying comorbidity and post-extubation injury [8, 12, 29]. The level of evidence of factor ‘underlying comorbidity’ comes from prospective and retrospective observational studies. Due to a critical risk of bias, mainly by confounding and selection bias, the level of evidence was downgraded resulting in a level of evidence of very low.
